# ALKBH5-mediated CHAC1 depletion promotes malignant progression and decreases cisplatin-induced oxidative stress in gastric cancer

**DOI:** 10.1186/s12935-023-03129-9

**Published:** 2023-11-25

**Authors:** Chunting Chen, Ertao Zhai, Yinan Liu, Yan Qian, Risheng Zhao, Yan Ma, Jianqiu Liu, Zhixin Huang, Jianhui Chen, Shirong Cai

**Affiliations:** 1 Division of Gastrointestinal Surgery Center, The First Affiliated Hospital of Sun Yat-sen University, Guangzhou, 510080 Guangdong P. R. China; 2 Gastric Cancer Center, Sun Yat-sen University, Guangzhou, 510080 Guangdong P. R. China; 3 Laboratory of Surgery, The First Affiliated Hospital, Sun Yat-Sen University, No.58, Zhong Shan Er Lu, 510080 Guangzhou, P. R. China

**Keywords:** M6a, ALKBH5, Oxidative stress, Gastric cancer

## Abstract

The m6a demethyltransferase ALKBH5 dynamically modulates gene expression and intracellular metabolic molecules by modifying RNA m6a in cancer cells. However, ALKBH5’s function in gastric cancer (GC) has remained controversial. This study demonstrates that ALKBH5 is highly expressed in GC. Silencing ALKBH5 hampers proliferation, and metastatic potential, and induces cell death in GC cells. Through a comprehensive analysis of the transcriptome and m6A sequencing, alterations in certain ALKBH5 target genes, including CHAC1, were identified. ALKBH5’s demethylation effect regulates CHAC1 RNA stability, leading to reduced CHAC1 expression. Moreover, CHAC1 modulates intracellular ROS levels, influencing the chemotherapy sensitivity of gastric cancer. In summary, our study unveils the pivotal role of the ALKBH5-CHAC1-ROS axis and highlights the significance of m6A methylation in gastric cancer.

**Supplementary Information:**

The online version contains supplementary material available at 10.1186/s12935-023-03129-9.

## Introduction

Gastric cancer (GC) ranks as the fifth most prevalent tumor and the third most fatal cancer globally. Nearly half of all gastric cancer cases worldwide are diagnosed in East Asia [1, 2]. The majority of gastric cancer patients receive their diagnosis during advanced stages of malignant proliferation and metastasis [3]. Such late-stage diagnoses often result in grim prognoses [4, 5]. Hence, it is vital to identify new biomarkers and therapeutic targets to enhance the diagnosis and treatment of GC.

Maintaining intracellular antioxidant levels is considered a distinctive feature of cancer [6, 7]. Cancer cells’ ability to maintain intracellular antioxidant levels is a distinctive characteristic. They regulate oxidation reduction levels, resisting damage from chemotherapeutic agents and reactive oxygen species (ROS) [8, 9]. Reactive oxygen species (ROS; H_2_ O_2_; O_2_^−^; OH^−^ etc.) are produced by aerobics; and cause extensive damage to cellular components under stress and injury conditions [10–12]. In cancer cells, the production of intracellular ROS are increased significantly due to mitochondrial dysfunction [13], metabolic changes [14], and frequent genetic mutations [15]. As a result, cancer cells derive a series of adaptive responses to counteract the level of ROS in *vivo*. Glutathione (GSH), a vital antioxidant, acts as a scavenger and detoxifier of oxygen free radicals [16]. Under oxidative stress, GSH reacts with ROS and is converted to GSSG by GSH-dependent peroxidases [17]. GSH plays a dual role in cancer progression which is essential for the removal and detoxification of carcinogens. However, elevated GSH levels protect tumor cells against chemotherapeutic agents in bone marrow, breast, colon, laryngeal, and lung cancers [8, 9, 18]. Thus, understanding the molecular mechanisms behind altered redox and oxygen radicals in GC is important for future diagnostic and therapeutic strategies.

N6-methyladenosine (m6A) is the most prevalent internal modification of mRNA in eukaryotes [19], which participates in many aspects of in *vivo* regulation, including regulation of mRNA stability, splicing, translocation, localization, and translation [20]. M6A modifications which are installed by methyltransferases (METTL3, METTL14) and removed by RNA demethylases (FTO, ALKBH5) [21] are widely investigated in cancers. In recent studies, m6A has been reported to be associated with stem cell differentiation, chemotherapy resistance, and tumor progression [21]. In gastric cancer, METTL3 has been reported to promote cell proliferation and metastasis through different downstream genes [22–24]. While another methylase METTL14 inhibited tumor progression via miR-30c-2-3p/AKT1S1/circORC5 axis [25]. Nevertheless, the biological significance of ALKBH5 has been contentious in various studies. ALKBH5 regulates lncRNA TP53TG1 and NEAT1, promoting tumor proliferation and metastasis [26, 27]. Conflicting evidence showed that ALKBH5 suppressed tumor invasion through PKMYT1 [28]. In this study, we elucidate ALKBH5’s role in GC progression. Mechanistically, ALKBH5 downregulates CHAC1 expression by eliminating m6A modification, disrupting ROS homeostasis in gastric cancer.

## Results

### Elevated ALKBH5 expression is associated with poor prognosis in gastric cancer patients

**Fig. 1:**
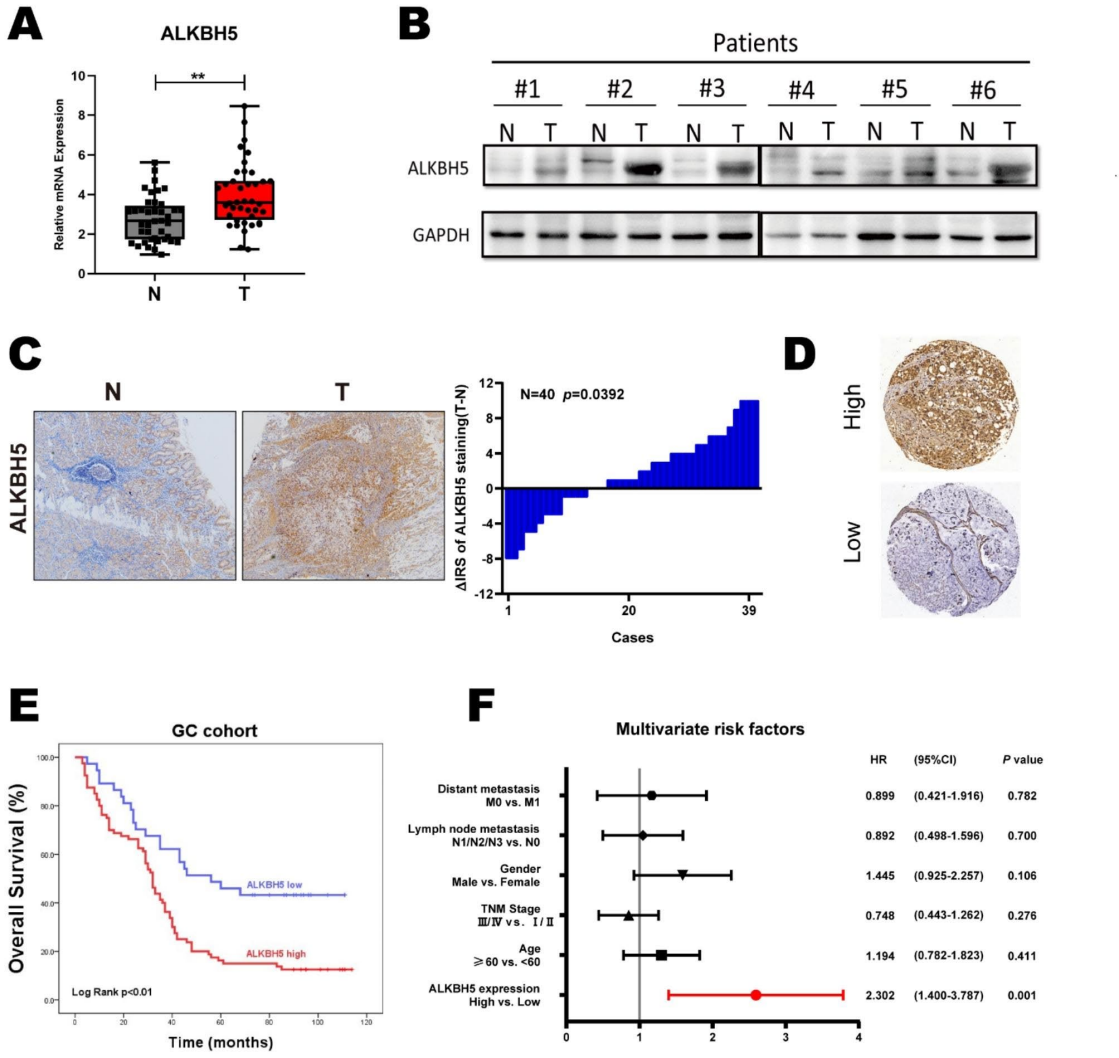
ALKBH5 is associated with poor prognosis in gastric cancer. (**A**) Expression levels of ALKBH5 in gastric cancer and paired normal gastric mucosal tissues were detected using qRT-PCR (n = 40). (**B**) ALKBH5 protein levels in GC tissues and paired normal gastric mucosal tissues were detected by western blotting (n = 6). (**C**) Representative IHC images were displayed with anti-ALKBH5 antibody (labeled bars were 200 μm) (left).Differential distribution of ALKBH5 immunoreactivity score (IRS) (ΔIRS = IRST-IRSN). (n = 40)(right). (**D**) Representative IHC images of tissue microarrays were displayed with anti-ALKBH5 antibody. (**E**) IRS scoring was performed using tissue microarrays (TMA) with anti-ALKBH5 antibody probes, and the mean value was taken as the cut-off value, followed by Kaplan-Meier OS analysis of ALKBH5 expression in gastric cancer patients (n = 117, *p* < 0.01, log-rank test). (**F**) Multivariate analysis was performed in the GC cohort. All bars correspond to 95% CIs. Data are expressed as mean ± SD. * *p* < 0.05, ** *p* < 0.01 and ∗∗∗ *p* < 0.001 (one-way ANOVA; t-test). All assays were repeated biologically 3 times

In the public database (http://gepia.cancer-pku.cn/index.html), ALKBH5 expression was significantly elevated in gastric cancer (GC) tissues compared to normal tissues (Figure S1A). Using Kaplan-Meier analysis (https://kmplot.com/analysis/), we observed that patients with gastric cancer who had higher ALKBH5 mRNA levels exhibited poorer progression-free survival (PFS) and overall survival (OS), especially among male patients (Figures S1B, C). We then investigated ALKBH5 expression in both normal and gastric cancer tissues from our clinical center using RT-qPCR, western blotting, and immunohistochemical (IHC) analyses. The results consistently showed a significant elevation in ALKBH5 levels (Fig. 1A-C). Furthermore, we confirmed that gastric cancer patients with elevated ALKBH5 expression had poorer OS among our patients (n = 117, *p* < 0.01, log-rank test; Fig. 1D-E). Multiple Cox regression analysis revealed that ALKBH5 was an independent risk factor for poor prognosis in gastric cancer patients (HR = 0.434, 95% CI (0.264–0.714); Fig. 1F). Taken together, these findings suggest that ALKBH5 is upregulated in gastric cancer and indicates a worse prognosis for patients with GC.

### ALKBH5 promoted the progression of gastric cancer in vitro and in vivo

**Fig. 2:**
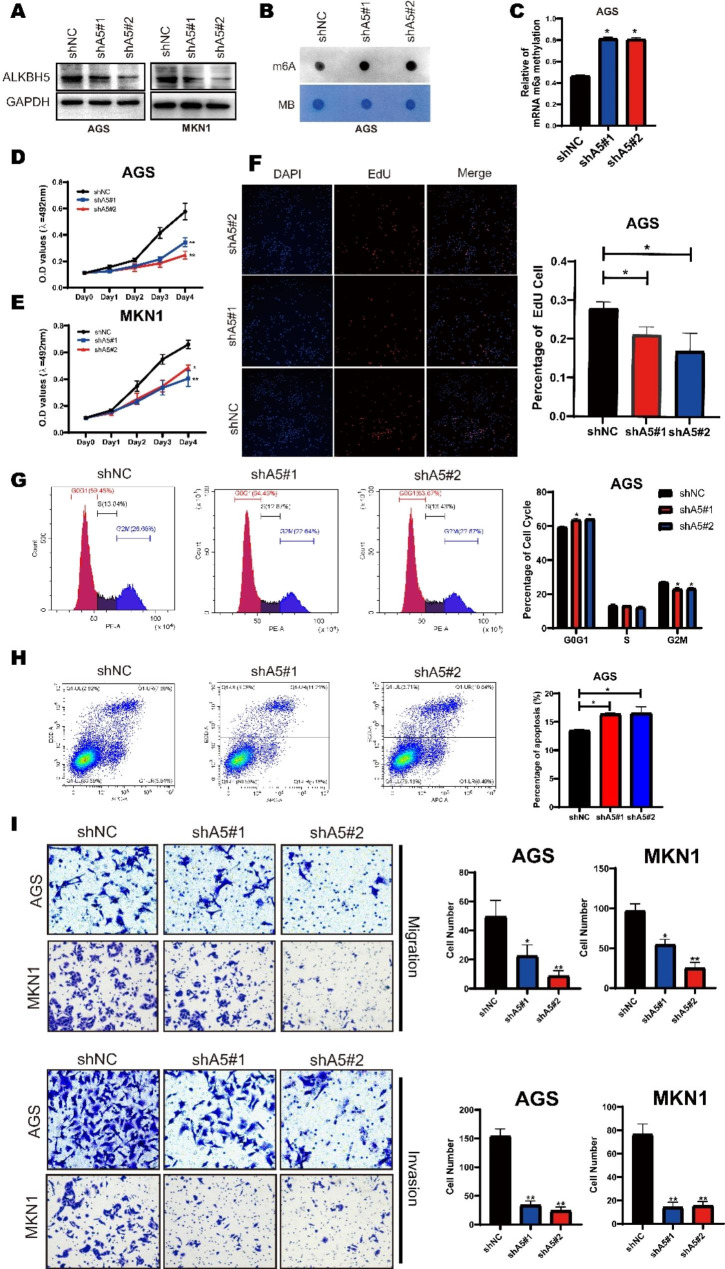
ALKBH5 affects gastric cancer cell progression in multiple ways. (**A**) Western blotting of ALKBH5 protein levels in MKN1 (right) and AGS (left) cells after ALKBH5 knockdown. (**B**) mRNA isolated from GC cells with knockdown of ALKBH5 was analyzed by spot hybridization with m6A antibody. MB (methylene blue) staining was used as a control. (**C**) Through EpiQuik M6A RNA Methylation Quantification Kit colorimetric method to detect the m6a level from the model. (**D**-**E**) MTT Cell Proliferation and Cytotoxicity Assay Kit for two ALKBH5 knockdown sequences and control in AGS and MKN1 cells. (**F**) ALKBH5 knockdown and control AGS cells were stained with azide 594 (red) to detect EdU and DAPI (blue) to stain cell nuclei. Fluorescence images were obtained and analyzed by fluorescence microscopy (left). Values are expressed as mean ± SD compared to the control group, n = 3 * *p* < 0.05 (right). (**G**) Cell cycle analysis using propidium iodide (PI) staining of ALKBH5 knockdown and control AGS cells. (left): Representative images. (right): Quantitative data. (**H**) ALKBH5 knockdown and apoptosis analysis using membrane coupling protein **V**/propidium iodide (PI) staining in control AGS cells. (left): Representative images. (right): Quantitative data. (**I**) Transwell cell migration (upper) analysis of ALKBH5 knockdown and control groups in AGS and MKN1 cells. Matrigel matrix gel invasion assay (bottom) of ALKBH5 knockdown and control groups in AGS and MKN1 cells. Left: Representative images. Right: quantitative data. Data are expressed as mean ± SD. * *p* < 0.05, ** *p* < 0.01 and ∗∗∗ *p* < 0.001 (one-way ANOVA; t-test). shA5-1, shALKBH5-1; sh A5-2, sh ALKBH5-2; shNC, negative control shRNA. all in vitro assays were repeated biologically 3 times

To detect the function of ALKBH5 in GC, we established stable knockdown, wild type, and mutant H204A overexpression of ALKBH5 in GC cell lines through ALKBH5 baseline expression (Fig. 2A and Figure S2A-C). The m6A level on altering ALKBH5 expression was verified by dot blot and m6A RNA Methylation Quantification Kit. We found that m6a levels were significantly increased in ALKBH5-depleted cells, and conversely decreased after overexpression of wild type, but not mutant ALKBH5 (Fig. 2B, C and Figure S2D, E). CCK-8 assay suggested that cell growth of AGS and MKN1 was dramatically inhibited after ALKBH5 knockdown (Fig. 2D, E). Similar results were obtained from the EdU cell proliferation assay, in which the proportion of EdU + cells declined after the knockdown of ALKBH5(Fig. 2F). We next examined the cell cycle by flow cytometry, and it showed that the proportion of cells in the G2/M phase significantly decreased in ALKBH5-deplete cells (Fig. 2G). Conversely, ALKBH5 overexpression promoted cell proliferation and increased the proportion of G2/M in GC cell lines (Figure S2F-I). AnnexinV/ PI staining showed that ALKBH5 knockdown led to a lower proportion of apoptotic cells in GC, and the opposite effect was observed after overexpression (Fig. 2H, Fig S2J). Apart from these, the Transwell assay revealed the inhibition of migration and invasion ability of ALKBH5 knockdown (Fig. 2I), While overexpression of wild type, but not mutant ALKBH5 had the opposite effect (Figure S2K). Moreover, the EMT pathway was induced by ALKBH5 overexpression and inhibited by ALKBH5 knockdown (Figure S3).

**Fig. 3:**
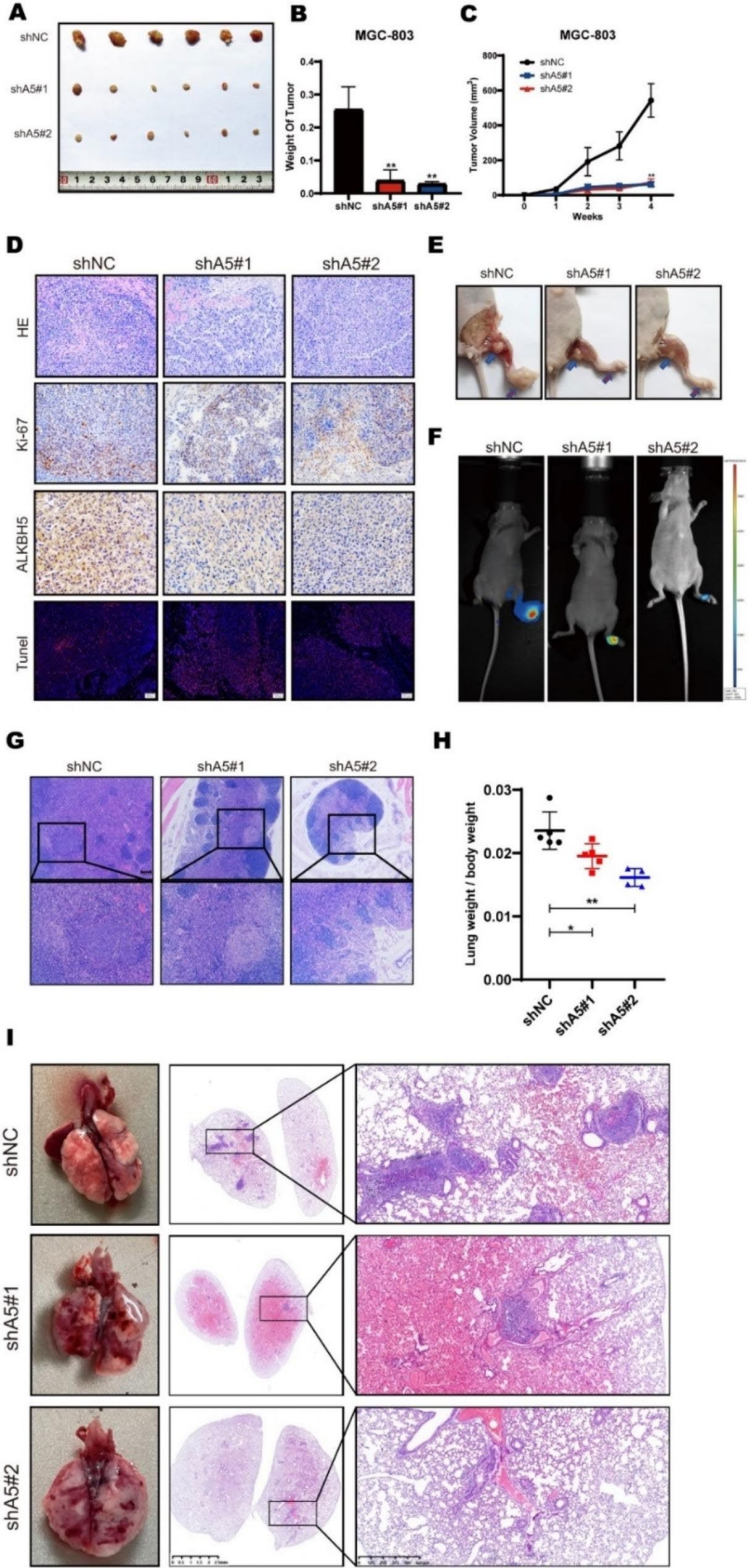
In vitro, ALKBH5 further stimulates the malignant progression of gastric cancer. (**A**) Subcutaneous implantation of ALKBH5 knockdown and control MGC-803 cells (n = 6) in GC mouse tumor. Two sequences were used in this experiment. (**B**) Comparison of tumor weight between GC mice implanted with ALKBH5 knockdown and control MGC-803 cells. (**C**) Comparison of tumor growth volume between GC mice implanted with ALKBH5 knockdown and control MGC-803 cells. (**D**) Sections of subcutaneous transplanted tumors from nude mice transfected with ALKBH5 knockdown were stained with HE (top), immunohistochemical staining (middle) (scale bar = 100 μm), and detected with antibodies against Ki67 and ALKBH5 (middle panel) Tunel Apoptosis Assay Kit (bottom panel). (**E**) Mice footpad implanted with ALKBH5 knockdown and control MGC-803 cells (n = 6) in GC mice (red arrow: mouse footpad tumor in situ; blue arrow: mouse popliteal metastatic lymph node swelling). (**F**) Mice with foot pad lymphatic metastasis model in vivo fluorescence imaging system. (**G**) Metastatic lymph nodes of nude mice transfected with ALKBH5 knockdown were sectioned and stained with HE (Upscale bar = 100 μm; Down: scale bar = 40 μm) for comparison of lymph node metastasis rate, and representative images were selected for each group. (**H**) Comparison of lung weights divided mice weights of GC mice implanted with ALKBH5 knockdown and control MGC-803 cells. (**I**) Representative images of lungs from GC mice with tail vein injection of ALKBH5 knockdown and control MGC-803 cells (n = 6; left). Lung sections of nude mice transfected with ALKBH5 knockdown were stained with HE (left panel: scale bar = 2.5 mm; right panel: scale bar = 625 μm) to compare the rate of bloodstream metastasis of gastric cancer, and representative images of lungs were selected for each group (right). Data are expressed as mean ± SD. * *p* < 0.05, ** *p* < 0.01 and ∗∗∗ *p* < 0.001 (one-way ANOVA; t-test). shA5-1, shALKBH5-1; sh A5-2, sh ALKBH5-2; shNC, negative control shRNA. all in vivo assays were repeated 3 times biologically

Later, we were concerned whether ALKBH5 had the same effect in *vivo*. First, we further validated the in *vivo* oncogenic function of ALKBH5 using a subcutaneous model in nude mice (Fig S4A-C). Compared to the control group, mice injected with ALKBH5-depleted GC cells showed significantly slower tumor growth, which was reflected in the reduction of tumor size and weight (Fig. 3A-C). In addition, IHC experiments showed that the proportion of ALKBH5 and tumor proliferation marker Ki-67 decreased in ALKBH5 knockdown tissues and the proportion of TUNEL-positive cells was increased in the knockdown groups (Fig. 3D). Furthermore, we constructed the mouse footpad lymphatic metastasis model to detect lymph metastatic ability (Fig. 3E). After six weeks of injection, popliteal lymph node metastasis was validated by bioluminescent assays (Fig. 3F). Compared with the control, there was less lymph node metastasis in the ALKBH5 knockdown group (Fig. 3G). Finally, we injected the cells from the tail vein of the mouse to establish the lung metastasis mouse model (Figure S4D). After six weeks, the lung weight of ALKBH5 knockdown groups was lower than that of the control group (Fig. 3H). In addition, the number of tumor-forming lung lesions was also decreased in ALKBH5 knockdown groups (Fig. 3I). Overall, our results revealed that ALKBH5 promoted GC progression both in *vitro* and in *vivo*.

### Analysis of downstream targets of ALKBH5 in GC

**Fig. 4:**
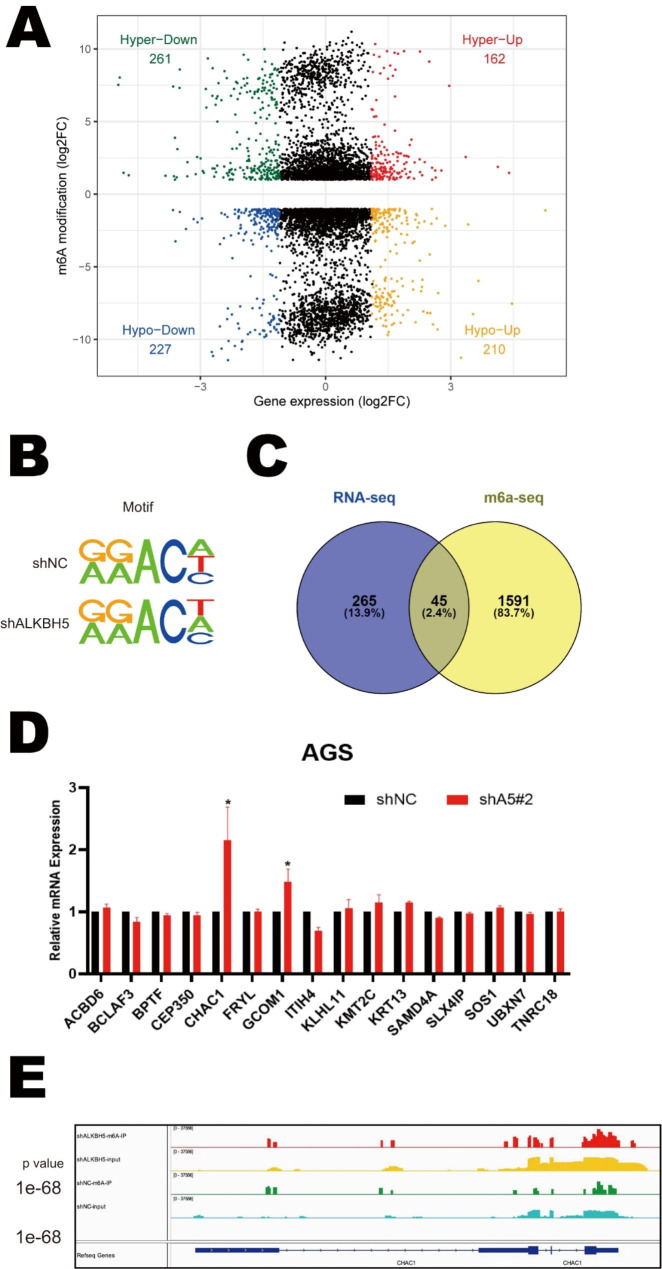
Exam the downstream targets of ALKBH5 in Gastric Cancer. (**A**) Extraction of MeRIP-seq data comparing the distribution of genes with differential expression and degree of m6a modification in AGS cells with those in the control group. (**B**) HOMER highest shared motifs with m 6 A-seq peaks in AGS cells with or without ALKBH5 knockdown. (**C**) In the RNA-seq dataset (m 6 A-seq input library) we selected 265 genes that showed more than 8-fold numerical differences. In shALKBH5 there are 5,180 unique m 6 A peaks for 1591 genes. The two were intersected and 45 genes were identified. (**D**) RT-qPCR was used to detect the expression of the 16 genes screened out in ALKBH5 knockdown versus control cells (Data are expressed as mean ± SD. * *p* < 0.05), and assays were repeated 3 times biologically. (**E**) MeRIP-seq data comparing the expression of the m 6 A-seq peak at the CHAC1 locus in ALKBH5 knockdown and control cells

To investigate the m6a modification function of ALKBH5 in GC, we performed Me-RIP and total RNA sequencing using ALKBH5 knockdown GC cells and its negative control to observe the difference in m6A modification of RNAs and gene expression profiles on ALKBH5 knockdown (Fig. 4A). We mapped the m6A-seq independent biological replicates and revealed that gGAC motifs were highly enriched at the m6A site in GC cells (Fig. 4B). There were 18,848 and 18,283 m6A peaks in the control and ALKBH5-deficient cells respectively. Among the GC cells with shALKBH5 knockdown, 5180 new peaks appeared and 5745 peaks disappeared. The other 13,103 peaks were found in both knockdown and control cells (Fig S5A). Since ALKBH5 is an m6A demethylase, we focused on those genes whose m6a modifications were increased after the knockdown of ALKBH5.

Subsequently, to investigate whether the knockdown-specific peaks are associated with differentially expressed genes in transcriptomics. We compared the two sequencing data to screen out the genes that performed the major functions. In the RNA-seq dataset, we selected 265 genes showing more than 8-fold numerical variation. Next, in these 265 genes, 45 genes were identified gaining m6A peaks on ALKBH5 knockdown (Fig. 4C). A preliminary GO enrichment analysis of 45 genes was performed using the online biometric analysis tool (http://metascape.org/ )which revealed that these genes were mainly associated with mRNA metabolic processes and DNA damage pathways (Fig S5B, C). Finally, 16 genes, including ACBD6, BCLAF3, BPTF, CEP350, CHAC1, FRYL, GCOM1, ITIL4, KLHL11, KMT2C, KRT13, SAMD4A, SLX4IP, SOS1, UBXN7, and TNRC18, which are associated with DNA damage or malignant phenotype, were selected for further validation. Among these 16 genes, only CHAC1 and GCOM1 were found to be dramatically elevated on ALKBH5 knockdown in GC cells, while CHAC1 was the most pronounced (Fig. 4D). Moreover, the m6a-specific peak was significantly higher in the ALKBH5 knockdown group than in the control group at the CHAC1 locus (Fig. 4E). Thus, we hypothesized that CHAC1 was the downstream target of ALKBH5 regulating GC progression.

### ALKBH5 suppressed CHAC1 expression via interfering RNA stability

**Fig. 5:**
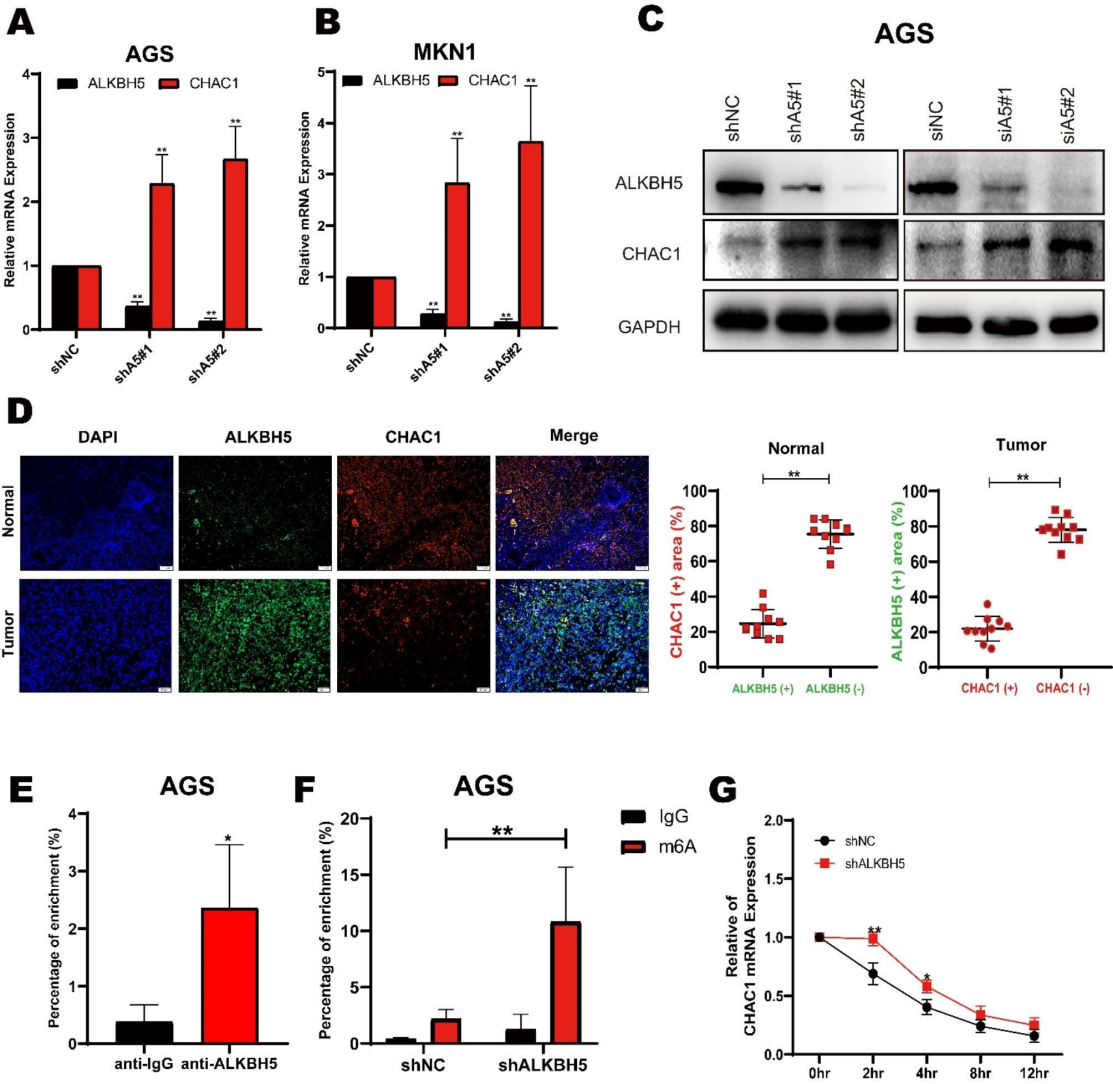
ALKBH5 regulates the expression of CHAC1 in GC. (**A**) Expression of two ALKBH5 knockdown sequences in AGS cells versus control for CHAC1 by RT-qPCR. (**B**) Expression of two ALKBH5 knockdown sequences in MKN1 cells was detected by RT-qPCR with CHAC1 expression in the control group. (**C**) Western blot detection of overexpression (left), sh knockdown (middle), and si knockdown (right) of CHAC1 and GCOM1 in AGS cells. (**D**) Correlation between ALKBH5 and CHAC1 protein expression in GC specimens., representative IF images of n = 10 GC specimens. Scale bar, Normal: 100 μm; Tumor: 50 μm. Fluoroscopic areas were calculated by Imaging J in 5 randomly selected microscopic fields for each cancer and paracancer specimen. t test calculates the percentage of ALKBH5 positive areas compared to ALKBH5 negative areas in CHAC1 positive areas; the percentage of CHAC1 positive areas compared to CHAC1 negative areas in ALKBH5 positive areas. Lines show the mean and standard deviation. (**E**) RIP (RNA immunoprecipitation) analysis of ALKBH5 enrichment of CHAC1 mRNA in AGS cells. (**F**) MeRIP-qPCR analysis of CHAC1 mRNA in AGS cells with or without ALKBH5 knockdown at m6A levels. (**G**) qPCR of CHAC1 mRNA stability in AGS cells with or without ALKBH5 knockdown. Identical amounts of RNA from cells treated with 2 μg/ml actinomycin D for 0 to 12 h were collected and measured by qPCR

To evaluate if ALKBH5 suppressed CHAC1 expression steadily in GC, we re-examined the total mRNA expression of CHAC1 after ALKBH5 knockdown in several gastric cancer cell lines. Consistent with the RNA sequencing, both MKN1 and AGS cells with ALKBH5 knockdown showed an increase in CHAC1 mRNA expression (Fig. 5A-B). Western blotting analysis also confirmed that both transient and stable ALKBH5 knockdown elevated the protein abundance of CHAC1 (Fig. 5C). Immunofluorescence experiments showed that ALKBH5 and CHAC1 expression was increased consistently in GC tissues compared to adjacent mucosa tissues (Fig. 5D). Additionally, we performed an analysis to investigate the correlations between these two genes within the TCGA gastric cancer dataset, revealing noteworthy negative associations (Figure S5D). Next, we investigated whether CHAC1 was the direct substrate of ALKBH5. To determine this, we conducted an RNA immunoprecipitation (RIP) experiment. This technique aims to identify RNA sequences that interact with a specific RNA-binding protein of interest in vivo. In our case, we needed to confirm whether the ALKBH5 protein could interact with CHAC1. The RIP analysis was carried out using ALKBH5 and a negative IgG antibody. RT-qPCR performed on the enriched products revealed a significantly higher enrichment of CHAC1 mRNA with the ALKBH5 antibody compared to the negative IgG control (Fig. 5E). Moreover, we investigated the link between CHAC1 and m6A using anti-m6A antibodies. Following the Me-RIP analysis, a significant increase in m6A modification on CHAC1 mRNA was confirmed after ALKBH5 knockout (Fig. 5F). These results suggest that ALKBH5 demethylates the m6A modification on CHAC1 mRNA, thereby suppressing its expression.

Subsequently, we explored the mechanism underlying ALKBH5-suppressed CHAC1 expression. ALKBH5 affects mRNA output and RNA metabolism [29]. Given that ALKBH5 knockdown elevated CHAC1 expression in both RNA and protein levels, we evaluated the stability of CHAC1 mRNA by measuring the loss of CHAC1 mRNA after blocking RNA synthesis with actinomycin D. The result showed that ALKBH5 knockdown was effective in maintaining CHAC1 mRNA stability (Fig. 5G). On the other hand, the stability of CHAC1 mRNA was significantly decreased after overexpression of ALKBH5 compared with the control group (Fig S5E). Overall, these results suggest that ALKBH5 demethylated the m6A modification on CHAC1 mRNA decreasing RNA stability to suppress CHAC1 expression in GC.

### ALKBH5 mediated CHAC1 regulated malignant progression of GC

**Fig. 6:**
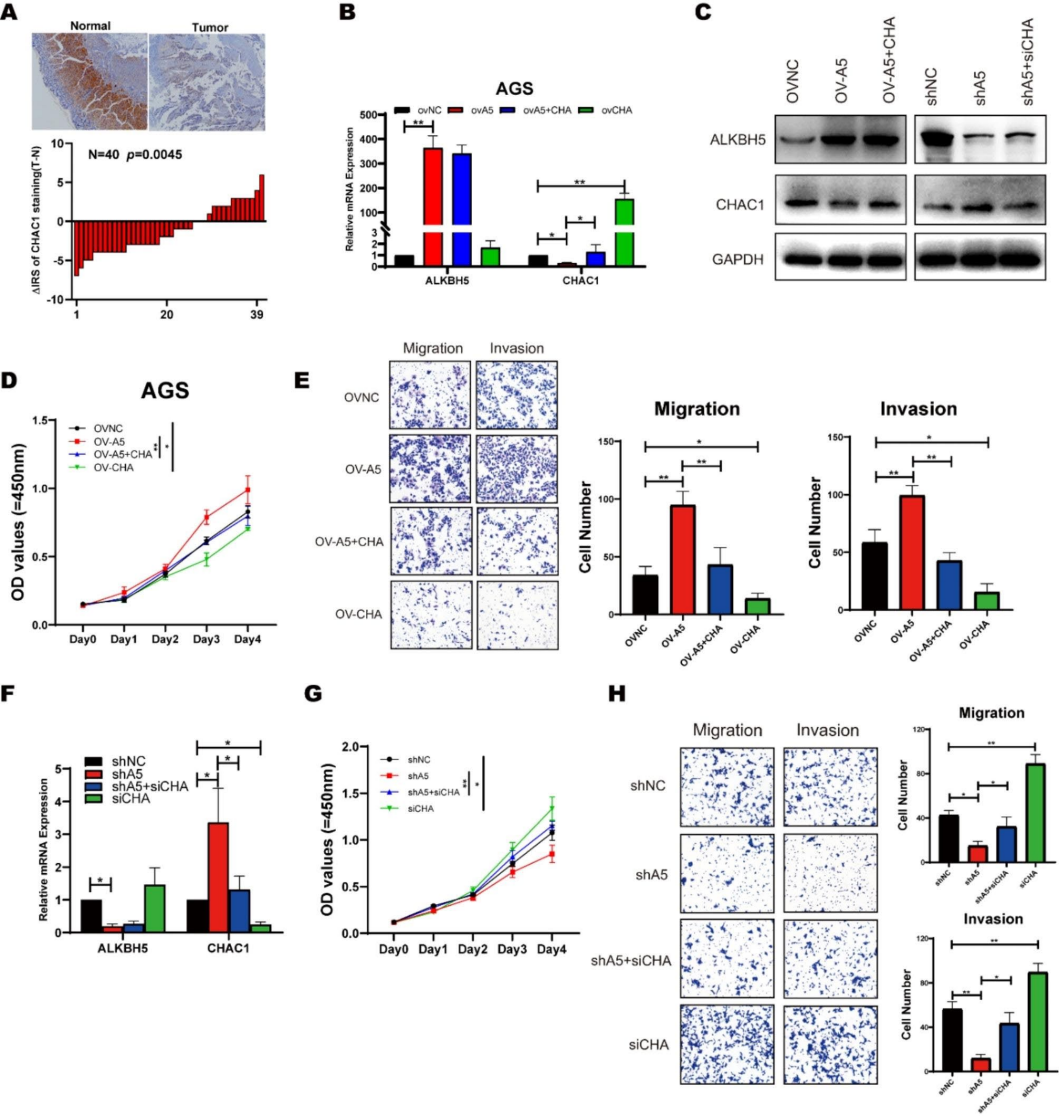
ALKBH5 regulates the expression of CHAC1 in gastric cancer, leading to modifications in proliferation migration and invasion. (**A**) Representative IHC images were displayed with anti-CHAC1 antibody (labeled bars were 200 μm) (upper).Differential distribution of ALKBH5 immunoreactivity score (IRS) (ΔIRS = IRST-IRSN). (n = 40, bottom). (**B**) Changes in mRNA levels of ALKBH5 and CHAC1 after simultaneous overexpression of ALKBH5 and CHAC1 in AGS cells were detected by RT-qPCR. (**C**) Changes in the protein levels of ALKBH5 and CHAC1 after simultaneous knockdown of ALKBH5 and CHAC1 in AGS cells (right); after simultaneous overexpression of ALKBH5 and CHAC1 (left) were detected by Western Blot. (**D**) Changes in cellular value-added function were detected after simultaneous overexpression of ALKBH5 and CHAC1 by applying a CCK8 reagent kit. (**E**) Application of Transwell and Matrigel to detect changes in cell migration and invasion functions in AGS cells that overexpress both ALKBH5 and CHAC1. (Left) Representative images; (right) Quantitative data. (**F**) Changes in mRNA levels of ALKBH5 and CHAC1 after simultaneous knockdown of ALKBH5 and CHAC1 in AGS cells were detected by RT-qPCR. (**G**) Changes in cellular value-added function were detected after simultaneous knockdown of ALKBH5 and CHAC1 by applying a CCK8 reagent kit in AGS cells. (**H**) Application of Transwell and Matrigel to detect changes in cell migration and invasion functions in AGS cells that knockdown both ALKBH5 and CHAC1. (Left) Representative images; (right) Quantitative data

CHAC1 is a member of the coding γ-glutamyl-transferase protein family, which plays a role in the regulation of glutathione levels and oxidative homeostasis in cells. In the TCGA database, CHAC1 indicated a better prognosis for GC patients (Figure S5F). Additionally, CHAC1 expression was suppressed in tumor tissues compared to adjacent normal tissues in our clinical samples (Fig. 6A, Figure S6A). To verify whether CAHC1 was the downstream gene of ALKBH5 regulating GC progression, we constructed a CHAC1 knockdown cell line to validate its biological role in GC (Fig S5B). Knocking down CHAC1 promoted proliferation and metastasis abilities, elevated GSH levels, and decreased ROS in GC (Figure S5C-G). Also, we established subcutaneous tumor models in nude mice. To ascertain whether CHAC1 plays a predominant role in ALKBH5-mediated gastric cancer proliferation, we generated two mouse models overexpressing these genes. Mice injected with MGC-803 control cells developed gastric cancer similar to that observed in humans, whereas overexpression of ALKBH5 significantly enhanced tumor formation (Figure S6H). Within 6 weeks of tumor formation, both overexpression models exhibited significant changes in tumor volume and weight (Figure S6I-J). We confirmed that ALKBH5 overexpression within tumor lesions increased the expression of the proliferation marker Ki67 while reducing CHAC1 expression (Figure S6K). Additionally, substantial inhibition of tumor growth was observed with CHAC1 overexpression (Figure S6I-J), accompanied by a significant decrease in Ki67 expression within the tumor (Figure S6K). Moreover, there was a significant difference in the Tunel cell proportion, a marker that detects DNA breaks formed during the final stages of apoptosis, between the two experimental groups (Figure S6K). Consequently, these findings underscore the anti-neoplastic role of CHAC1 in gastric cancer.

Besides, the proliferative capacity, that had been enhanced by overexpression of ALKBH5, was attenuated by re-expression of CHAC1 (Fig. 6B-D). The same trend was seen in the migration assay and the Matrigel matrix gel invasion assay. (Fig. 6E). In the ALKBH5 knockdown group, a consistent trend was also demonstrated, where the loss of malignant phenotypes after deletion of ALKBH5 was enhanced after the knockdown of CHAC1 (Fig. 6F-H). These results indicated that ALKBH5 inhibited CHAC1 expression to promote GC progression.

### ALKBH5-CHAC1 axis conferred the resistance to platinum-induced ROS of GC

Platinum-based chemotherapy is currently an important treatment for GC, which induces intracellular ROS synthesis. The GSH mechanism of drug resistance in common cancers such as ovarian cancer [30]and melanoma [31] has been described in previous reports. As CHAC1 plays an important role in the intracellular regulation of GSH and ROS, we explored whether the ALKBH5-CHAC1 axis affected platinum-induced ROS and platinum resistance of GC.

**Fig. 7:**
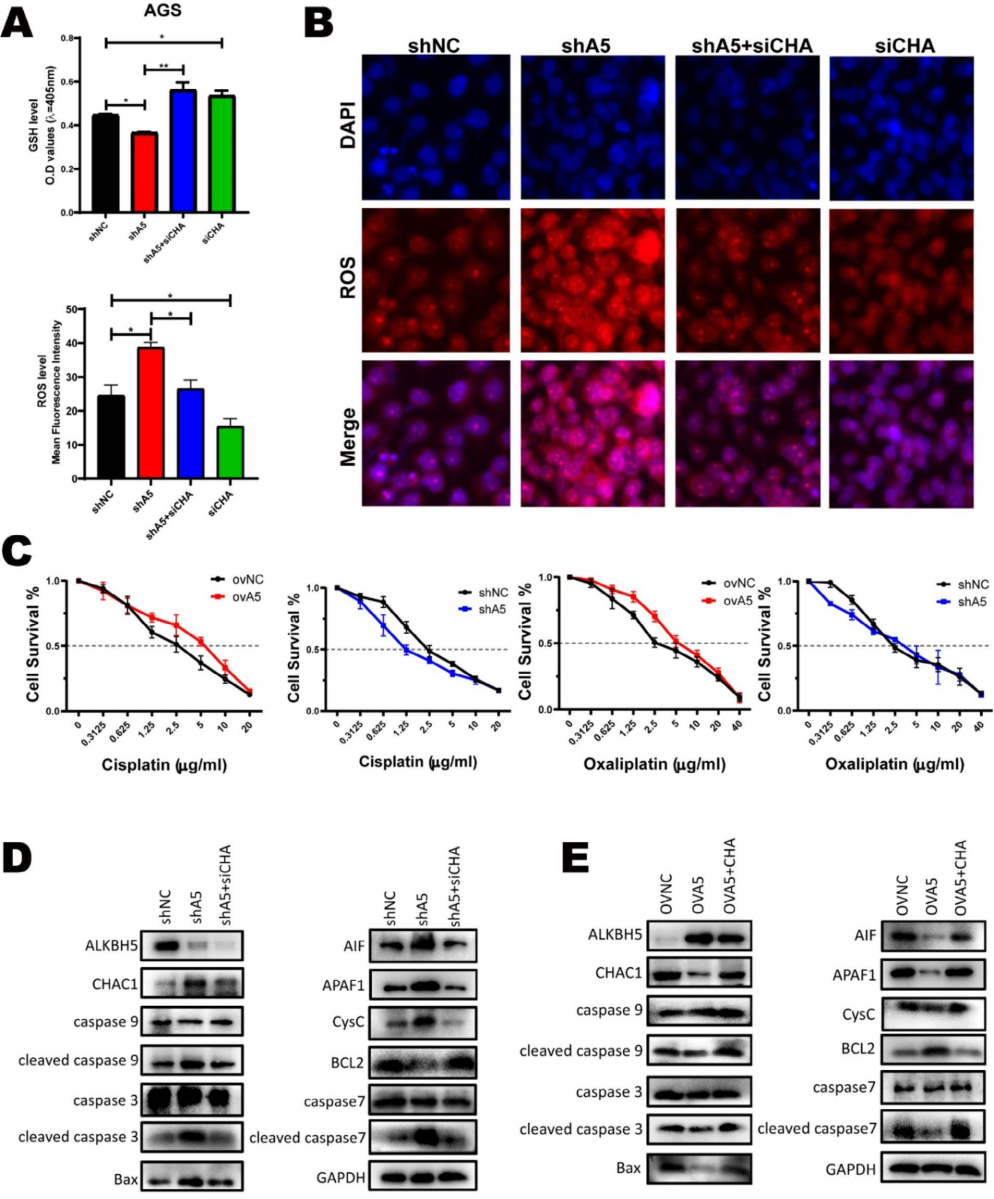
CHAC1 regulates the progression of ALKBH5 in GC and resisting cisplatin-induced ROS. (**A**) The total glutathione reagent kit was used to detect changes in GSH levels after treatment with knockdown of ALKBH5 and CHAC1. The average fluorescence intensity of each group in Fig. 7B was calculated by Image J software. (**B**) After treatment with 5ug/ml cisplatin for 36 h, the ROS probe was combined with the knockdown treated cells and the changes in ROS content were observed under the fluorescence microscope. (**C**) Cell survival was observed in the overexpression, knockdown, and control groups at different cisplatin(left), Oxaliplatin(right) concentrations, using a cell counting kit (CCK8) in MGC-803 cell line. (**D**) Western Blot was applied to detect changes in protein levels of markers commonly found in the mitochondrial apoptotic pathway in AGS cells with knockdown of ALKBH5 and CHAC1. (**E**) Western Blot was applied to detect changes in protein levels of markers commonly found in the mitochondrial apoptotic pathway in AGS cells with overexpression of ALKBH5 and CHAC1

Firstly, we found that the intracellular ROS levels were significantly increased when treated with cisplatin or oxaliplatin (Figure S6A, B). Then the GSH and ROS levels were investigated after ALKBH5 and CHAC1 expression changed by plasmids or siRNAs. The ELISA assays showed that the GSH level was decreased on ALKBH5 knockdown and further increased when we impaired CHAC1 expression with siRNA in ALKBH5 knockdown cells (Fig. 7A). The fluorescence image suggested that the intracellular ROS had an inverse trend to the GSH level (Fig. 7B). Correspondingly, the changing of GSH and ROS levels showed an opposite tendency on ALKBH5 and CHAC1 overexpression cells (Figure S6C, D). Next, we detected if the changing of GSH and ROS determined the platinum resistance of GC. Cell viability assay revealed that ALKBH5 overexpression enhanced the cell resistance to cisplatin and oxaliplatin, while ALKBH5 knocked down sensitive GC cells to platinum (Fig. 7C).

It is well known that an increase in ROS can lead to mitochondrial apoptosis [13]. Western blotting analysis verified that knocking down ALKBH5 upregulated the expression of cytochrome C and apoptosis-inducing factor (AIF) in AGS cells. In addition, protein levels of apoptosis genes (including cleaved caspase9, cleaved caspase7, cleaved caspase3, and BAX) were correspondingly upregulated. In contrast, the expression of BCL2, a suppressor of apoptosis, was downregulated. These changes were restored after the knockdown of CHAC1 (Fig. 7D). Opposite changes in the apoptosis pathway were noted in overexpressed ALKBH5 and CHAC1 (Fig. 7E). In conclusion, the ALKBH5-CHAC1 axis controlled intracellular GSH and ROS to confer platinum resistance of GC.

## Discussion

In human RNA, m6a is considered to be the most prevalent modification among the more than 100 chemical modifications [19, 21]. A series of recent studies have shown that m6A modifications are involved in a variety of human diseases, including type II diabetes, cancer progression, viral infections, and heart failure [32–34]. M6A modifications are dynamically regulated through m6A methyltransferases, demethylases, and readers, which modulate the biological functions of RNA. The m6A writer, METTL3, have been described to promote the malignant progression of GC in different pathway [22–24]. Taken for granted, the demethylase ALKBH5 was thought to be an antioncogene for GC. However, more evidence suggested that the function of ALKBH5 in GC was much more dependent on its downstream targets [27, 28]. In our study, we found that ALKBH5 was upregulated in tumors compared to adjacent normal tissues. High ALKBH5 expression indicated worse long-term outcomes in GC patients. In addition, in *vitro* and in *vivo* studies revealed that ALKBH5 promotes tumor growth, distance metastasis, and suppressed cell death through its m6A catalytic activity. Therefore, we hypothesized that ALKBH5 promoted tumor malignant progression of GC in a common environment.

CHAC1 encodes γ-glutamylcyclo transferase 1, a member of the transferase protein family [35]. Previous studies reported that the suppression of the ATF4-CHOP-CHAC1 pathway protected dendrite cells from ferroptosis [36]. In kidney renal and gastric cancer, CHAC1 could act as a biomarker to predict the long-term survival of patients [37, 38]. Biological function analysis suggested that CHAC1 is involved in the glutathione cycle inducing ER stress and cell death [39, 40]. Currently, research on CHAC1 in gastric cancer is not widely explored, Hui-Hwa Tseng et al. revealed that the Loc100506691-CHAC1 axis may play a key coordination role in metformin-induced tumor growth suppression [41]. In the Tomohisa Ogawa et al. study they found that overexpressed CHAC1 in H. pylori-infected parietal cells may cause the H. pylori-induced somatic mutations that contribute to the development of gastric cancer [42]. These studies also suggest that CHAC1 might contribute to the development of gastric cancer. In our study, we present additional evidence through the integration of RNA-seq and MeRIP-seq data, identifying CHAC1 as a potential downstream target of ALKBH5 in gastric cancer (GC). Our research demonstrates that ALKBH5-mediated m6A demethylation leads to CHAC1 mRNA degradation. By conducting CHAC1 knockdown experiments, we have confirmed that CHAC1 plays a role in suppressing tumor growth and metastasis in GC. Overexpression CHAC1 attenuated the malignant progression caused by ALKBH5 overexpression. Subsequently, as CHAC1 is involved in the glutathione cycle controlling intracellular ROS homeostasis, we measured the GSH and ROS levels to verify ALKBH5-CHAC1 axis influenced peroxidation balance in GC.

Platinum-based chemotherapy is the first-line treatment strategy for advanced GC patients. In response to chemotherapeutic agents, the corresponding reactive oxygen species, such as ROS, are reactively increased. It has been reported that moderate ROS levels can support survival and proliferation by activating signaling pathways that contribute to tumor growth in the tumor microenvironment [43, 44]. However, excessive ROS accumulation, failure of proper scavenging mechanisms, or lack of antioxidants can lead to severe damage to biomolecules, which can trigger cell death, including cancer cells [11, 12, 14]. Through a series of validations, we found that the ALKBH5/CHAC1/GSH axis has a role in regulating the level of ROS and apoptosis in cancer cells. In addition, ALKBH5 reduced the sensitivity of GC cells to cisplatin and oxaliplatin. These data provide a novel potential target for covering the chemotherapy resistance of GC.

In conclusion, our findings reveal the oncogenic role of ALKBH5 in GC development. Mechanistically, ALKBH5 demethylates m6A modifications of CHAC1 mRNA suppressing CHAC1 expression. Furthermore, the ALKBH5 / CHAC1/ROS axis promotes GC tumorigenesis and metastasis by altering oxidative levels. Therefore, ALKBH5 may be a potential predictor and therapeutic target for gastric cancer.

## Method details

### Cell lines and cell culture

AGS 、MKN1、MKN28、MGC-803、HGC-27 human gastric cancer cell lines and GES-1 human gastric epithelial cell lines were obtained from the Cell Bank of the Type Culture Collection of the Chinese Academy of Sciences (Shanghai, China). The cells were incubated in RPMI 1640/DMEM (Gibco BRL, Grand Island, NY) with 10% fetal bovine serum, and maintained in a 37 °C incubator with 5% CO_2_.

### Cell viability assay

Cell Counting Kit-8 assays were performed to assess cell viability. Briefly, cells were grown in 96-well plates and incubated for 24 h at a density of 1.0 × 10^4^ cells per well. After treatment with BD for 24 or 48 h, the cells were incubated for an additional 4 h with 100 μl of RPMI 1640/DMEM and 10 μl of CCK-8 solution at 37 °C. The absorbance of each well was then read at 450 nm using a microplate reader (Bio-Rad, Model 680, USA).

### Clinical tissue specimens

A total of 40 surgical resection specimens of gastric cancer patients from the Gastrointestinal Surgery Centre of the First Affiliated Hospital of Sun Yat-sen University were collected, including one copy each of cancer and precancerous tissues for subsequent experimental analysis. Inclusion criteria: (1) patients with a pathological diagnosis of gastric adenocarcinoma, (2) patients who underwent radical surgery for gastric cancer at the Gastrointestinal Surgery Centre, (3) patients with complete prognostic follow-up data, (4) patients who had never received any chemotherapy or molecular targeted therapy before surgery. Exclusion criteria: (1) patients with a pathological diagnosis of squamous carcinoma or other non-gastric adenocarcinoma, (2) patients with other malignancies in combination, (3) incomplete medical records and prognostic follow-up information, (4) patients who have received neoadjuvant chemotherapy or molecular targeted therapy before surgery. Fresh tissue specimens removed during surgery were stored in liquid nitrogen for quick freezing. They were used for subsequent pathological sectioning; protein and RNA extraction; sequencing analysis, etc. Patients’ consent was obtained for the use and collection of all tissue specimens, and the ethics committee of the First Affiliated Hospital of Sun Yat-sen University approved it.

### Tissue microarrays (TMA) sample

A total of 117 surgical resection specimens from gastric cancer patients at the Gastrointestinal Surgery Centre of the First Affiliated Hospital of Sun Yat-sen University were collected. Each specimen was prepared as cancer tissues on one glass slide for subsequent experimental analysis, adhering to the same criteria as described above. These specimens were utilized for subsequent pathological sectioning and analyzed these factors with K-M survival and multivariate analysis, we followed up with these patients from May 2004 to December 2011, during which we set a code of 1 for death and 0 for survival. More detailed clinical information of these patients is provided in Table S1. Patient consent was obtained for the collection and use of all tissue specimens, and the ethics committee of the First Affiliated Hospital of Sun Yat-sen University approved the study.

### Apoptosis and cell cycle analysis by flow cytometry

After transfection for 48 h, the gastric cancer cell population was treated with serum-free medium after starvation for 24 h at a density of approximately 70% in 6-well plates. Cells were harvested with EDTA-free trypsin and washed 2 times with PBS (2000 rpm, 5 min). Cells were then stained using the AnnexinV-AF647/PI kit (ES Science, China) according to the kit’s instructions. Finally, the samples were analyzed by flow cytometry.

### Western blot analysis

Cell lysates were separated by SDS-PAGE (7–12%) at 120 V and electro transferred onto nitrocellulose membranes (Millipore). After blocking with 5% nonfat dry milk in PBS, the membranes were incubated with the primary antibodies at 4 °C overnight and with horseradish peroxidase (HRP)-conjugated secondary antibodies for 1 h at room temperature. GAPDH and β-actin were used as controls.

### H&E staining

Tissue samples from the mice were fixed with formalin and embedded in paraffin. After cutting into 4 μm sections, the tumor, and essential organ specimens were stained with H&E, and histological examinations were performed using an Olympus microscope (Japan).

### Measurement of intracellular ROS

2′,7′-Dichlorodihydrofluorescein diacetate was used to detect intracellular ROS levels. After incubation with DHE for 24 h, the cells were washed with PBS and incubated in a fresh medium containing DCFH-DA (10 μg/ml) at 37 °C for 30 min. Then, the cells were collected and analyzed at an excitation wavelength of 488 nm and an emission wavelength of 610 nm. Images were taken with Olympus Upright Microscope.

### Immunofluorescence and immunohistochemistry

For immunohistochemistry (IHC) and immunofluorescence (IF) analysis, tissue sections of GC mouse xenografts or surgical specimens were dewaxed, rehydrated through an alcohol series, and then antigenically modified with sodium citrate, EDTA buffer solution. Tumor sections were blocked with 5% normal goat serum (Vector) in PBS containing 0.1% Triton X-100 and 3% H2O2 for 60 min at room temperature and then incubated overnight at 7 °C with the appropriate primary antibody. IHC staining was performed with horseradish peroxidase (HRP) coupling using DAB detection. IF staining was performed using the appropriate Alexa Fluor 488 or Alexa Fluor 594 secondary antibody (Invitrogen, diluted 1:1000). The image was taken with a Zeiss Axio Scope.A1 vertical microscope.

For IF analysis of cultured cells, GSC was fixed with 4% formaldehyde (Fisher) for 15 min and then blocked with 5% normal goat serum (carrier) in PBS with or without 0.1% Triton X-100 for 60 min at room temperature. Immunostaining was performed using appropriate primary and secondary antibodies. Nuclei were double-stained with DAP1. Images were taken with an Olympus vertical microscope.

### MeRIP-qPCR

MeRIP assay was adapted from a reported protocol [45]. Briefly, intact poly-A-purified RNA was denatured to 70 °C for 10 min, transferred immediately on ice, and then incubated with m^6^A antibody in 1ml buffer containing RNasin Plus RNase inhibitor 400 U (Promega), 50 mM Tris-HCl, 750 mM NaCl, and 0.5% (vol/vol) Igepal CA-630 (Sigma Aldrich) for 2 h at 4 °C. Dynabeads Protein G (Invitrogen) were washed, added to the mixture, and incubated for 2 h at 4 °C with rotation. m^6^A RNA was eluted twice with 6.7 mM N^6^-methyladenosine 5′-monophosphate sodium salt at 4 °C for 1 h and precipitated with 5 μg glycogen, one-tenth volumes of 3 M sodium acetate in 2.5 volumes of 100% ethanol at − 80 °C overnight. m^6^A enrichment was determined by qPCR analysis. Fragmented mRNA was directly incubated with m^6^A antibody-containing buffer and treated similarly. The eluted m6A mRNA fragments were concentrated for RNA-seq library construction. A KAPA Stranded mRNA‐seq Kit (F. Hoffmann‐La Roche Ltd, Grenzacherstrasse, Basel, Switzerland) was employed for the generation of RNA‐seq libraries for both m6A antibody‐enriched and input mRNAs (Illumina, San Diego, CA, USA). The resultant libraries were diluted to a final concentration of 8 pmol/L. Clusters were generated on an Illumina cBot using a HiSeq 3000/4000 PE Cluster Kit (#PE‐410‐1001, Illumina) and then subjected to sequencing on an Illumina HiSeq 4000 platform. Subsequently, all reads were aligned to the human genome 19 using BWA tools and annotated with miRbase version 21 (https://www.mirbase.org) and miRDeep2 (https://www.mdc-berlin.de). Data analysis was performed using R software (Free Software Foundation, Boston, MA, USA).

### RNA isolation and quantitative real-time PCR

RNA was isolated using TRIzol™ Reagent (Life Technologies) following the manufacturer’s protocol. cDNA was generated using the Master mix cDNA Synthesis Kit (TAKARA). Quantitative real-time PCR using Powerup SYBR Green PCR Master Mix (Life Technologies) was performed on a 7500 Fast Real-time PCR System (Roche LightCycle480). For RNA stability assay, GCs were plated in a poly-lysine coated 6-cm dish and incubated with actinomycin D (MCE) at 5 μg/ml for the indicated time. Total RNA was isolated for qPCR analysis. See Table S2 for quantitative PCR primers.

### m6A quantification

The change of global m^6^A levels in mRNA was measured by EpiQuik m^6^A RNA Methylation Quantification Kit (Colorimetric) (EpigenTek) following the manufacturer’s protocol. 200 ng poly-A-purified RNA was used for each sample analysis.

### EdU incorporation and cell-cycle analysis

For the EdU (5-bromo-2′-deoxyuridine) incorporation assay, cells were cultured with an EdU-labeling reagent (RiboBio) and stained with an anti-EdU antibody (Cell Signaling) according to the manufacturer’s instructions. Five fields of view per slide were examined for EdU-positive cells.

### Plasmids and RNA knockdown

ALKBH5 expression plasmid was generated by cloning the full-length ORF of the human ALKBH5 gene (NM_017758) into the pcDNA3.1-DYK vector (GeneCopoeia). ALKBH5 H204A was generated by GeneCopoeia. ALKBH5 and CHAC1 were cloned to the pcDNA3.1 vector without a tag. Wild-type and H204 mutants of ALKBH5 were cloned to pLVX (GeneCopoeia) for stable expression.

Transfections were performed using X-treme GENE HP DNA Transfection Reagent for plasmid and X-treme GENE siRNA Transfection Reagent (Roche) for siRNA following the manufacturer’s protocols. Briefly, 2 μg plasmids and 5 μL Lipofectamine 3000 were diluted in 150 μL Opti-MEM. The plasmids and Lipofectamine 3000 solution were mixed and incubated at room temperature for 15 min. Subsequently, the mixture was added to 6-well plates, and cells were collected after 48–72 h for further studies. As for siRNAs were diluted to 5 nmol/L in 100 μL Opti‐MEM (Gibco) and mixed with 5 μL Lipofectamine RNAi Max. Following a 20-minute incubation at room temperature, the mixture was introduced into the cell culture medium. After 48 h, the cells were harvested. The specific siRNA sequences and plasmids are detailed in Supplementary Table S1.

### TUNEL assay

Deparaffinize and rehydrate slides, Microwave antigen retrieval in microwave (600 ml of 10mM Na Citrate, pH 6) Cool 20´. Wash 3 × 5´ in water. Wash 1 × 5´ in 1x PBS (Phosphate-Buffered Saline). Shake off/wipe off excess PBS and circle all sections with ImmunoEdge or PAP pen. Block 10 min in Equilibration Buffer at room temperature (50 μl/section). Add 50 μl of Reaction Buffer to each section and incubate at 37 °C for 60–90 min. [One section with terminal deoxynucleotidyl transferase (TdT) enzyme, one section without enzyme as a negative control]. Soak slides in 1xSSC for 15 min at room temp to stop reaction. Wash 5 × 5´ in 1x PBS. Mount the sections in 3:1 Vectashield: DAPI. Coverslip and seal with clear nail polish.

### Lentiviral transduction for stable cell lines

Lentiviral vectors expressing non-targeting pLKO.1 control shRNA (SCH002), and two shRNA constructs targeting ALKBH5 (NM_017758), shRNA1 (TRCN0000064783) and shRNA2 (TRCN0000064787) were obtained from (GeneCopoeia). The lentiviral vectors were co-transfected with packaging vectors psPAX2 and pMD2G (Addgene) into 293Ta cells for lentivirus production. To establish stable cell lines, GC cells were transduced by using the above lentiviruses with polybrene (6 μg/ml, Sigma). After 72 h of transduction, cells were selected with 2 μg/ml puromycin for 4 days. For the ALKBH5 rescue experiment, shRNA targeting 3′UTR of ALKBH5 (shRNA1) was used for knockdown.

### Qualifications and statistical analysis

Data are presented as the mean ± standard error of the means (SEM), or standard deviations (SD). Differences in the mean values between the 2 groups were assessed for significance with a 2-tailed Student t-test using GraphPad Prism 6.0. Kaplan-Meier survival data were analyzed using the log-rank test. The Pearson correlation test was used to assess relationships between variables in tumor tissues.

### Electronic supplementary material

Below is the link to the electronic supplementary material.

Supplementary Material 1: (**A**): Via GEPIA database, the RNA experssion of ALKBH5 in gastric cancer is higher in tumor tissue compare with the normal tissue. (**B**) PFS Kaplan-Meier survival curves based on ALKBH5 expression using the online bioinformatics tool Kaplan-Meier Plotter https://kmplot.com/analysis/ (n = 522, logrank *p* = 0.015). (**C**) OS of male patients based on ALKBH5 expression using the online bioinformatics tool Kaplan-Meier Plotter https://kmplot.com/analysis/ (n = 349 ,logrank *p* < 0.001)

Supplementary Material 2: (**A**) Expression levels of ALKBH5 in normal gastric mucosal epithelial cells and each gastric cancer cells were detected by qRT-PCR. (**B**) The expression levels of ALKBH5 in normal gastric mucosal epithelial cells and each gastric cancer cell were detected by western blotting. (**C**) Validation of protein expression levels in ALKBH5 overexpression, mutation models in common gastric cancer cell lines. (**D**) mRNA isolated from GC cells with overexpression and mutation of ALKBH5 was analyzed by spot hybridization with m6A antibody. MB (methylene blue) staining was used as control. (**E**) Through EpiQuik M6A RNA Methylation Quantification Kit colorimetric method to detect the m6a level from the models. (**F**) MTT Cell Proliferation and Cytotoxicity Assay Kit for ALKBH5 overexpression, H204A mutation and control sequences and control in MKN1 cell line. **G**) MTT Cell Proliferation and Cytotoxicity Assay Kit for ALKBH5 overexpression, H204A mutation and control sequences and control in AGS cell line. (**H**) Cell cycle analysis using propidium iodide (PI) staining of ALKBH5 overexpression and control AGS cells. (Upper): Representative images. (Bottom): Quantitative data. (**I**) ALKBH5 overexpression and control AGS cells were stained with azide 594 (red) to detect EdU and DAPI (blue) to stain cell nuclei. Fluorescence images were obtained and analyzed by fluorescence microscopy (left). Values are expressed as mean ± SD compared to control group, n = 3 * *p* < 0.05 (right). (**J**) ALKBH5 overexpress and apoptosis analysis using membrane coupling protein V/propidium iodide (PI) staining in control AGS cells. (Left): Representative images. (Right): Quantitative data. (**K**) Transwell cell migration and invasion analysis of ALKBH5 overexpression H204A mutation and control groups in GC cells. Left: representative images. Right: quantitative data

Supplementary Material 3: Validation of target genes in the EMT pathway in ALKBH5 overexpressed and knockdown protein samples

Supplementary Material 4: (**A**) Western blotting of ALKBH5 protein levels in MGC-803 cells after ALKBH5 knockdown. (**B**) mRNA levels of ALKBH5 in MGC-803 cells after ALKBH5 knockdown were detected by qRT-PCR. (**C**) T A general view of the nude rat subcutaneous tumour model constructed over a period of about 6 weeks Control group(Upper);Knockdown group (bottom). (**D**) A lung metastasis model constructed over a 6 week period

Supplementary Material 5: (**A**) Relationship between groups presenting the presence or absence of RNA methylation modifications using a Venn diagram. Between groups, we defined peaks without overlap as peaks specific to the treatment group (shALKBH5: 5180; shNC:5745), and peaks with overlap as peaks shared between groups (13103). The number of common and unique peaks between groups was counted. (**B**) An initial Gene Ontology (GO) enrichment analysis of the 45 genes was conducted utilizing the online bioinformatics analysis tool available at http://metascape.org/. (**C**) In the MeRIP data, the Top 20 GO pathways were enriched with genes unique to ALKBH5 knockdown. (**D**) Examination of the correlation between mRNA expressions of CHAC1 and ALKBH5 in the TCGA STAD dataset. (**E**) qPCR of CHAC1 mRNA stability in AGS cells with or without ALKBH5 overexpressed. Identical amounts of RNA from cells treated with 2 μg/ml actinomycin D for 0 to 12 hr were collected and measured by qPCR. (**F**) OS Kaplan-Meier survival curves based on CHAC1 expression using the online bioinformatics tool Kaplan-Meier Plotter https://kmplot.com/analysis/ exclude GSE62254 (n = 592, logrank *p* < 0.01)

Supplementary Material 6: (**A**) CHAC1 protein levels in GC tissues and paired normal gastric mucosal tissues were detected by western blotting (n = 8). (**B**) Detection of mRNA levels of CHAC1 triple-si sequences by RT-qPCR. (**C**) Cytometric Kit-8 measurements for three CHAC1 knockdown sequences and control AGS cells (CCK8). (**D**) CHAC1 knockdown and apoptosis analysis using membrane coupling protein V/propidium iodide (PI) staining in AGS cells. (left): Representative images. (right): Quantitative data. (**E**) Representative images of Transwell cell migration and Matrigel matrix gel invasion assay in CHAC1 knockdown and control groups (left). Quantitative data of Transwell cell migration and invasion assay (right). (**F**) Glutathione (GSH) assay kit (colorimetric method) for two CHAC1 knockdown sequences and control AGS cells. (**G**) Measurement of intracellular ROS fluorescence intensity by reactive oxygen species (ROS) detection kit and flow cytometry in two CHAC1 knockdown sequences and control AGS cells. (**H**) Gross representation of two overexpressed nude mouse models, ALKBH5(middle); CHAC1(right). (**I**) Comparison of tumor weight between GC mice implanted with ALKBH5, CHAC1 overexpress and control MGC-803 cells. (**J**) Comparison of tumor growth volume between GC mice implanted with ALKBH5, CHAC1 overexpress and control MGC-803 cells. (**K**) Sections of subcutaneous transplanted tumors from nude mice transfected with ALKBH5, CHAC1 overexpression were stained with HE (top), immunohistochemical staining (middle) (scale bar = 100μm), and detected with antibodies against Ki67,CHAC1 and ALKBH5 (middle panel) Tunel Apoptosis Assay Kit (bottom panel)

Supplementary Material 7: (**A**) The ROS fluorescence intensity was measured by flow cytometry after cisplatin (5ug/ml) induction at 24hr and 48hr, respectively. (**B**) The ROS fluorescence intensity was measured by flow cytometry after Oxaliplatin (5ug/ml) induction at 24hr and 48hr, respectively. (**C**) The total glutathione reagent kit was used to detect changes in GSH levels after treatment with overexpression of ALKBH5 and CHAC1. The average fluorescence intensity of each group in FigS7D calculated by Image J software. (**D**) After treatment with 5ug/ml cisplatin for 36hr, the ROS probe was combined with the overexpress treated cells and the changes in ROS content were observed under the fluorescence microscope

Supplementary Material 8

Supplementary Material 9

## Data Availability

The data that support the findings of this study are available from the corresponding author upon reasonable request.
