# Supplementary figures and images for: ALKBH5-mediated CHAC1 depletion promotes malignant progression and decreases cisplatin-induced oxidative stress in gastric cancer
Source: Cancer Cell Int. 2023 Nov 25;23:293. doi: 10.1186/s12935-023-03129-9 (PMC10676604; doi:10.1186/s12935-023-03129-9)

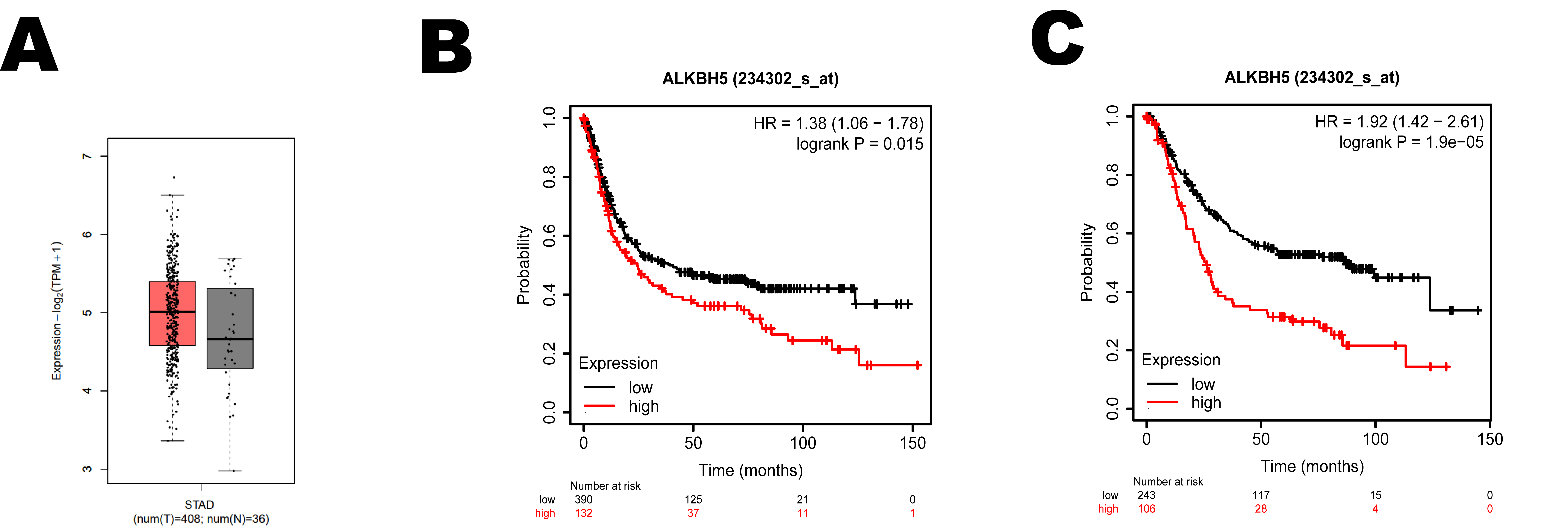

Supplement: Supplementary file 1 — Supplementary Material 1: (A): Via GEPIA database, the RNA experssion of ALKBH5 in gastric cancer is higher in tumor tissue compare with the normal tissue. (B) PFS Kaplan-Meier survival curves based on ALKBH5 expression using the online bioinformatics tool Kaplan-Meier Plotter https://kmplot.com/analysis/ (n = 522, logrank p = 0.015). (C) OS of male patients based on ALKBH5 expression using the online bioinformatics tool Kaplan-Meier Plotter https://kmplot.com/analysis/ (n = 349 ,logrank p < 0.001) [file 12935_2023_3129_MOESM1_ESM.png]

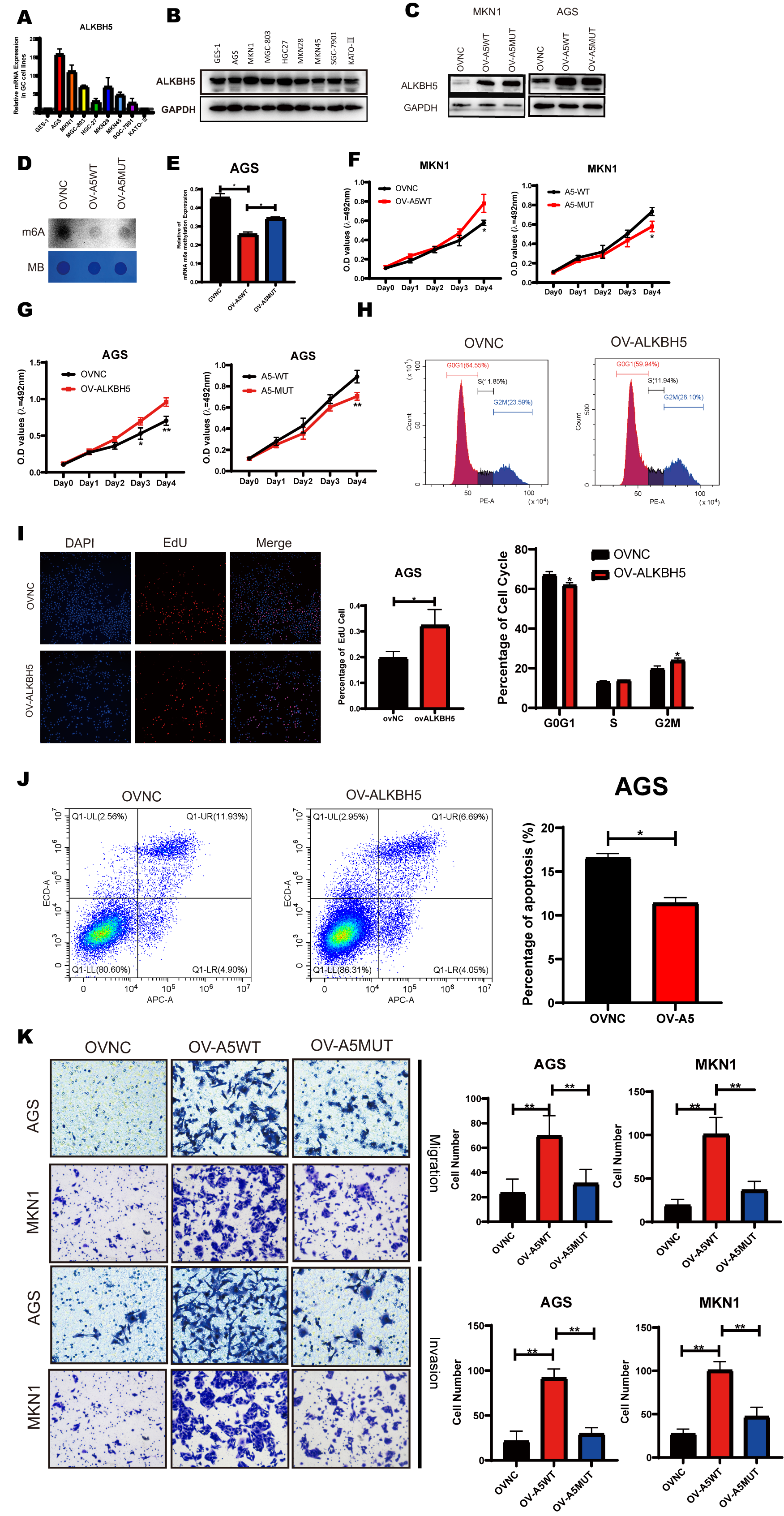

Supplement: Supplementary file 2 — Supplementary Material 2: (A) Expression levels of ALKBH5 in normal gastric mucosal epithelial cells and each gastric cancer cells were detected by qRT-PCR. (B) The expression levels of ALKBH5 in normal gastric mucosal epithelial cells and each gastric cancer cell were detected by western blotting. (C) Validation of protein expression levels in ALKBH5 overexpression, mutation models in common gastric cancer cell lines. (D) mRNA isolated from GC cells with overexpression and mutation of ALKBH5 was analyzed by spot hybridization with m6A antibody. MB (methylene blue) staining was used as control. (E) Through EpiQuik M6A RNA Methylation Quantification Kit colorimetric method to detect the m6a level from the models. (F) MTT Cell Proliferation and Cytotoxicity Assay Kit for ALKBH5 overexpression, H204A mutation and control sequences and control in MKN1 cell line. G) MTT Cell Proliferation and Cytotoxicity Assay Kit for ALKBH5 overexpression, H204A mutation and control sequences and control in AGS cell line. (H) Cell cycle analysis using propidium iodide (PI) staining of ALKBH5 overexpression and control AGS cells. (Upper): Representative images. (Bottom): Quantitative data. (I) ALKBH5 overexpression and control AGS cells were stained with azide 594 (red) to detect EdU and DAPI (blue) to stain cell nuclei. Fluorescence images were obtained and analyzed by fluorescence microscopy (left). Values are expressed as mean ± SD compared to control group, n = 3 * p < 0.05 (right). (J) ALKBH5 overexpress and apoptosis analysis using membrane coupling protein V/propidium iodide (PI) staining in control AGS cells. (Left): Representative images. (Right): Quantitative data. (K) Transwell cell migration and invasion analysis of ALKBH5 overexpression H204A mutation and control groups in GC cells. Left: representative images. Right: quantitative data [file 12935_2023_3129_MOESM2_ESM.png]

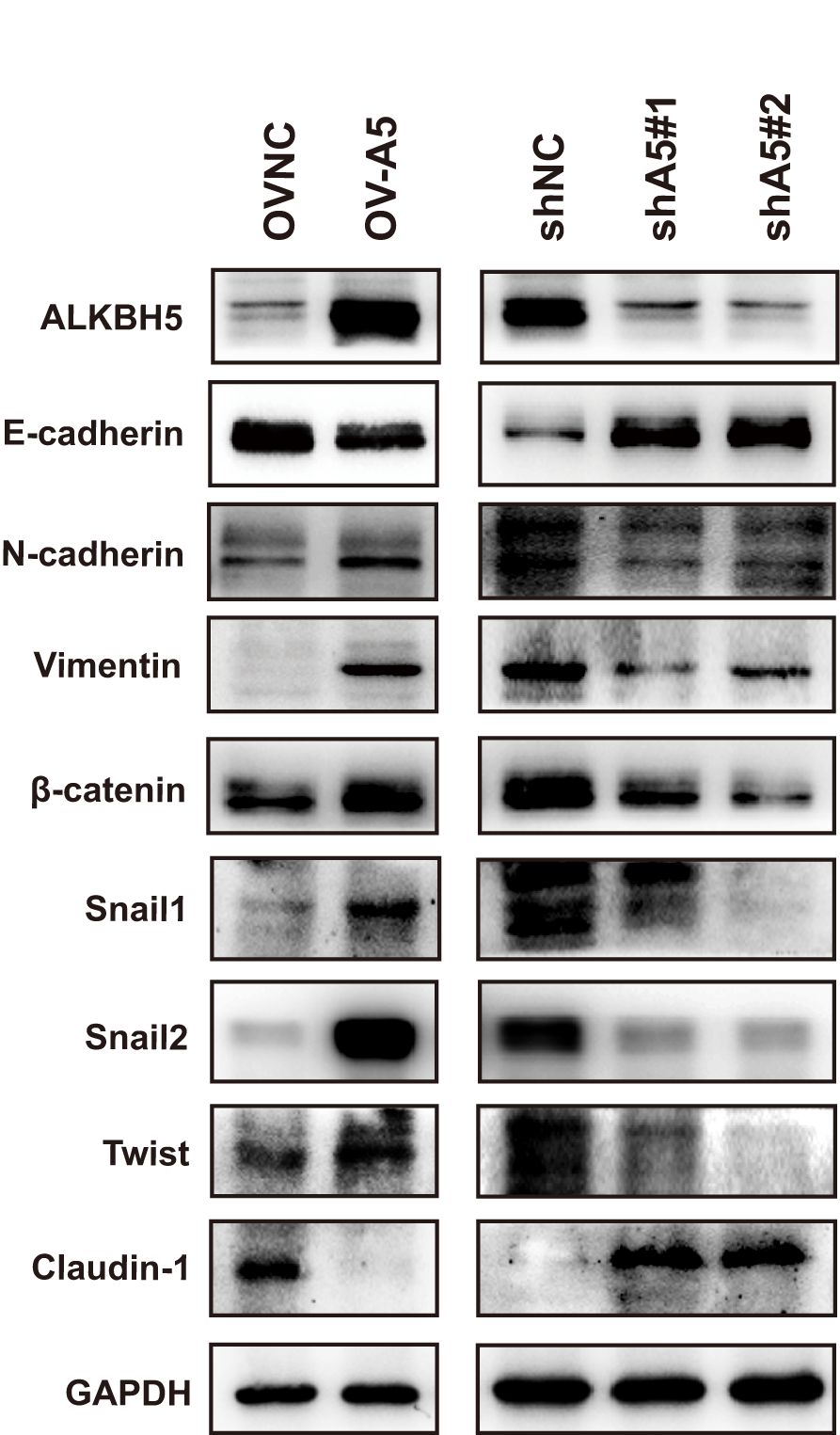

Supplement: Supplementary file 3 — Supplementary Material 3: Validation of target genes in the EMT pathway in ALKBH5 overexpressed and knockdown protein samples [file 12935_2023_3129_MOESM3_ESM.png]

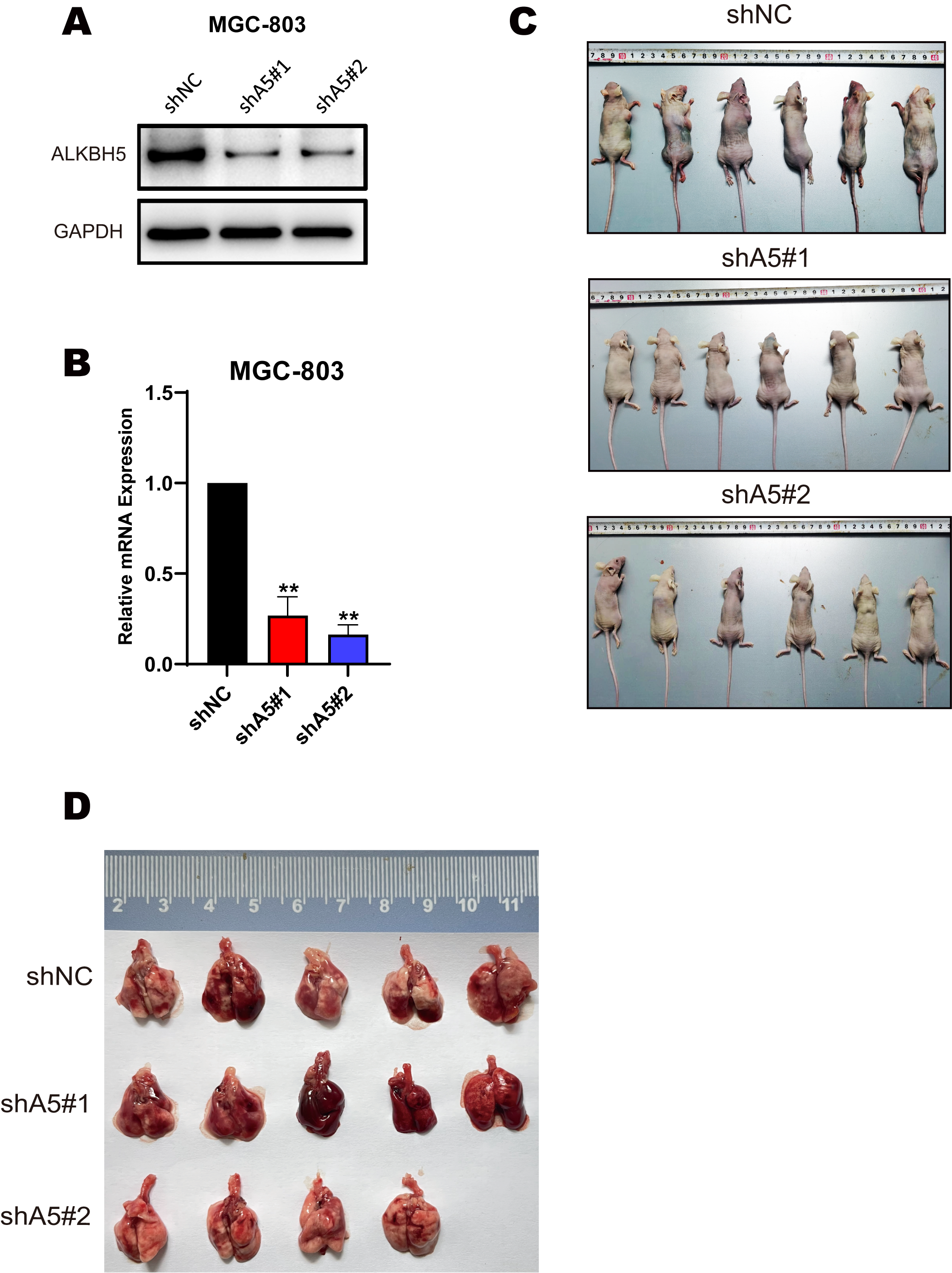

Supplement: Supplementary file 4 — Supplementary Material 4: (A) Western blotting of ALKBH5 protein levels in MGC-803 cells after ALKBH5 knockdown. (B) mRNA levels of ALKBH5 in MGC-803 cells after ALKBH5 knockdown were detected by qRT-PCR. (C) T A general view of the nude rat subcutaneous tumour model constructed over a period of about 6 weeks Control group(Upper);Knockdown group (bottom). (D) A lung metastasis model constructed over a 6 week period [file 12935_2023_3129_MOESM4_ESM.png]

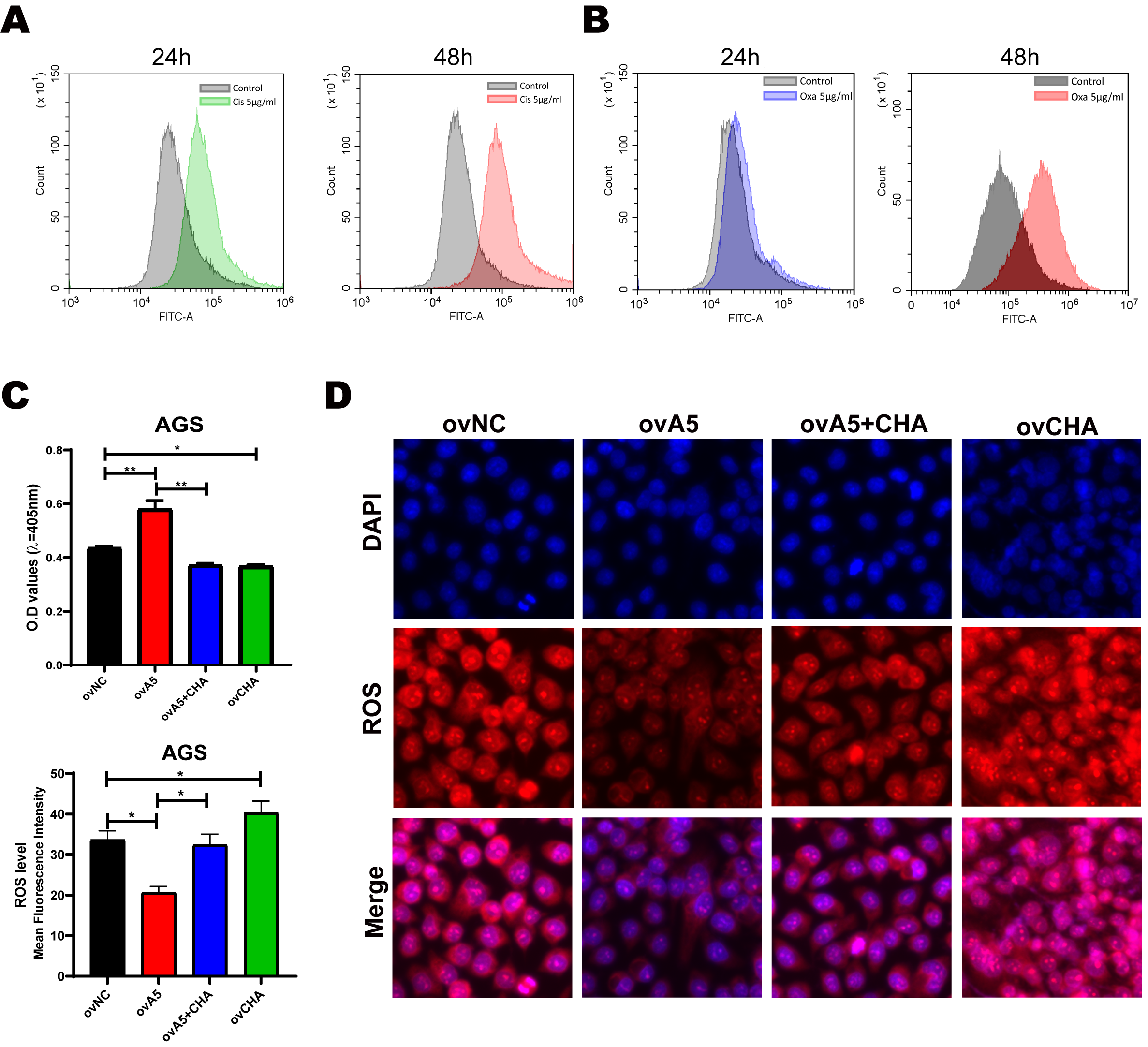

Supplement: Supplementary file 7 — Supplementary Material 7: (A) The ROS fluorescence intensity was measured by flow cytometry after cisplatin (5ug/ml) induction at 24hr and 48hr, respectively. (B) The ROS fluorescence intensity was measured by flow cytometry after Oxaliplatin (5ug/ml) induction at 24hr and 48hr, respectively. (C) The total glutathione reagent kit was used to detect changes in GSH levels after treatment with overexpression of ALKBH5 and CHAC1. The average fluorescence intensity of each group in FigS7D calculated by Image J software. (D) After treatment with 5ug/ml cisplatin for 36hr, the ROS probe was combined with the overexpress treated cells and the changes in ROS content were observed under the fluorescence microscope [file 12935_2023_3129_MOESM7_ESM.png]
